# Pneumococcal serotype determines growth and capsule size in human cerebrospinal fluid

**DOI:** 10.1186/s12866-020-1700-7

**Published:** 2020-01-20

**Authors:** Annelies Müller, Anke Salmen, Suzanne Aebi, Linda de Gouveia, Anne von Gottberg, Lucy J. Hathaway

**Affiliations:** 1 Institute for Infectious Diseases, Faculty of Medicine, University of Bern, Friedbühlstrasse 51, CH-3001 Bern, Switzerland; 2 Graduate School for Cellular and Biomedical Sciences, University of Bern, Bern, Switzerland; 3 Department of Neurology, Inselspital, University Hospital Bern and University of Bern, Bern, Switzerland; 4 National Institute for Communicable Diseases: Division of the National Health Laboratory Service, Johannesburg, South Africa; 5 Faculty of Health Sciences, School of Pathology, University of Witwatersrand, Johannesburg, South Africa

**Keywords:** *Streptococcus pneumoniae*, Human cerebrospinal fluid, Serotype, Capsule, Growth, Case fatality rate

## Abstract

**Background:**

The polysaccharide capsule is a major virulence factor of *S. pneumoniae* in diseases such as meningitis. While some capsular serotypes are more often found in invasive disease, high case fatality rates are associated with those serotypes more commonly found in asymptomatic colonization. We tested whether growth patterns and capsule size in human cerebrospinal fluid depends on serotype using a clinical isolate of *S. pneumoniae* and its capsule switch mutants.

**Results:**

We found that the growth pattern differed markedly from that in culture medium by lacking the exponential and lysis phases. Growth in human cerebrospinal fluid was reduced when strains lost their capsules. When a capsule was present, growth was serotype-specific: high carriage serotypes (6B, 9 V, 19F and 23F) grew better than low carriage serotypes (7F, 14, 15B/C and 18C). Growth correlated with the case-fatality rates of serotypes reported in the literature. Capsule size in human cerebrospinal fluid also depended on serotype.

**Conclusions:**

We propose that serotype-specific differences in disease severity observed in meningitis patients may, at least in part, be explained by differences in growth and capsule size in human cerebrospinal fluid. This information could be useful to guide future vaccine design.

## Background

*Streptococcus pneumoniae* is a leading cause of disease ranging from mild to severe manifestations. Invasive pneumococcal disease (IPD) includes the life-threatening conditions of bacteraemia and meningitis, both of which have high mortality rates [1, 2] and *S. pneumoniae* is also a leading cause of pneumonia. A major virulence factor of *S. pneumoniae* is the polysaccharide capsule and, based on the biochemical properties of the capsule, *S. pneumoniae* is categorized into different serotypes. Currently, approximately 100 serotypes are known [2, 3]. Several serotypes (including 7F, 14, 15B/C and 18C) have repeatedly been associated with invasive disease while other serotypes (such as 6B, 9 V, 19F and 23F) are more commonly associated with asymptomatic colonization of the human nasopharynx [1, 4–7]. There are also differences in clinical outcomes and mortality rates due to different serotypes [7–10]. The pneumococcal serotype 19F, for example, has repeatedly been associated with meningitis and a high case-fatality rate (CFR) [8, 9].

The pneumococcal capsule has been a target of research for several decades and current vaccines are composed of capsule polysaccharides. The most commonly used, pneumococcal conjugate vaccine 13 (PCV13) and pneumococcal polysaccharide vaccine 23 (PPSV23), contain serotype-specific polysaccharides for 13 and 23 serotypes respectively. The large-scale use of PCVs has led to changes in disease and carriage prevalence of individual serotypes over time and geographically [11–13]. These changes have increasingly led to non-vaccine type (NVT) serotypes emerging. For this reason, previous publications have stressed the need to assess and understand the invasive disease potential of both vaccine type (VT) and NVT serotypes to help guide future vaccine design [6, 9].

Serotypes differ in their ability to cause severe disease [14–18] and there is a correlation between polysaccharide production and case-fatality rates of serotypes in humans [19]. Pneumococcal strains with larger capsules are also more virulent in animal models [14, 20]. Previously we have shown that there is a link between serotype and capsule sizes in vitro in culture media and also between serotype and growth [21, 30]. In vitro studies with laboratory media such as brain heart infusion broth (BHI), show typical pneumococcal growth has an exponential and a lysis phase. The lysis phase has previously been reported to be due to cell death mainly induced by the pneumococcal autolysin LytA; see review [22]. Another study, however, showed that autolysis is caused by accumulation of hydrogen peroxide due to the expression of the *spxB* gene resulting in apoptosis [15].

Supporting the experimental findings, studies show that, although the outcome of pneumococcal disease can be affected by factors such as age, immunodeficiency and other host characteristics, even when controlling for these factors, certain serotypes are associated with more severe disease [16–18]. Similarly, other epidemiological studies have linked serotype to mortality rates in patients and to invasive disease potential [1, 7–9, 23].

It is not fully understood how the different serotypes are responsible for differences in disease severity. Therefore, here we studied the growth behaviour and capsule size of different serotypes in human cerebrospinal fluid (hCSF) rather than culture media to reflect more closely the environment the bacteria would encounter in a meningitis patient. Although other studies have looked at hCSF parameters in the setting of pneumococcal meningitis [24–27], data about the behaviour of *S. pneumoniae* in hCSF in vitro is scarce. We tested whether differences in ability to grow in hCSF in vitro and differences in capsule size correlate with serotype-specific disease severity.

## Results

### The growth pattern of *S. pneumoniae* in hCSF differs from that in culture medium

**Fig. 1:**
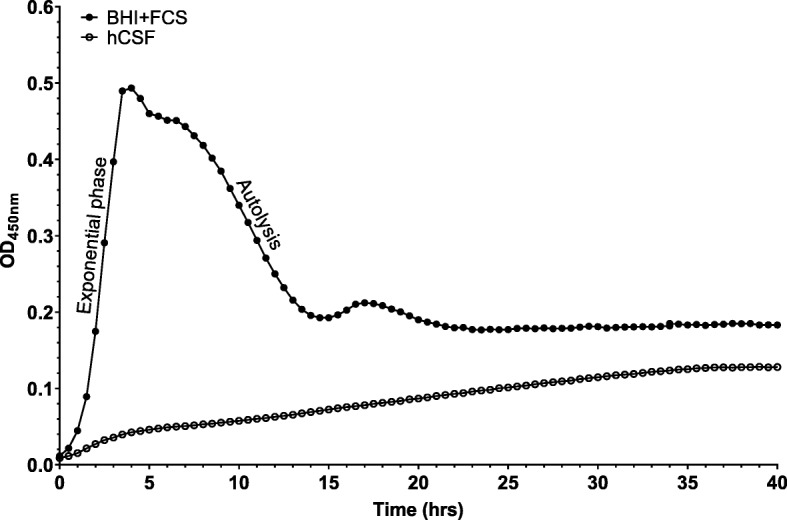
Growth pattern of *S. pneumoniae* wild type strain 106.66 in BHI + FCS and hCSF over 40 h. In BHI + FCS, 106.66 shows the typical exponential phase followed by a peak OD_450nm_ followed by an autolysis phase. In hCSF, 106.66 lacked the typical exponential phase, peak OD_450nm_ and the following autolysis phase. Each data point represents an average of 3 independent experiments

To determine whether *S. pneumoniae* is able to grow and how it behaves in hCSF in vitro, we performed growth analysis over 40 h of the strain 106.66 (serotype 6B) in BHI + FCS and hCSF. The growth pattern of strain 106.66 in hCSF lacked a discernible exponential phase and the autolysis phase typically seen when grown in BHI + FCS medium (Fig. 1). In hCSF, we observed an extended straight line of growth of 106.66 which plateaued after ~ 35 h (Fig. 1). This observation was consistent for 106.66 and all its capsule switch mutants which grew in hCSF (Additional file 1: Figure S2). No growth was observed in control wells containing hCSF with no inoculum (results not shown). To test whether this pattern of growth was due to limited nutrition, the bacteria were grown in CDM. The OD_max_ in CDM was noticeably lower than in BHI + FCS but the exponential phase and the autolysis phase were still present (Additional file 1: Figure S3). To test whether a factor in hCSF was inhibiting growth, the same strain was grown in a 1:1 mixture of BHI + FCS and hCSF. The growth in the 1:1 mixture did not differ from that in BHI + FCS (Additional file 1: Figure S3).

### Capsule aids growth in hCSF

**Fig. 2:**
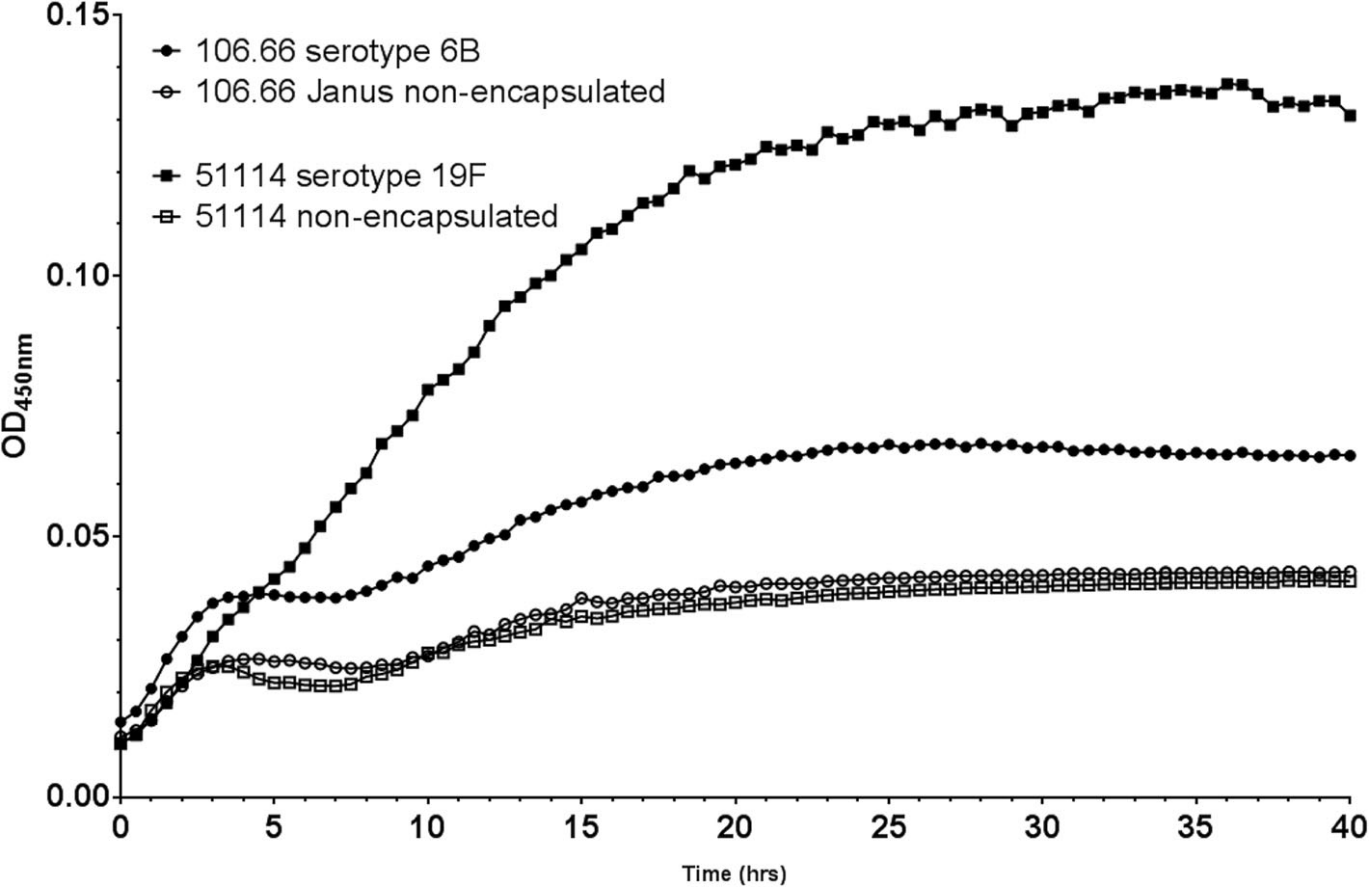
Growth of *S. pneumoniae* strains with and without capsule in hCSF over 40 h. Strain 106.66, its capsule deletion mutant (106.66 Janus) and the clinical isolate 51,114 with and without capsule (51,114 nonencapsulated) in hCSF over 40 h. Each data point represents an average of 3 independent experiments

To determine the effect of capsule on growth of *S. pneumoniae* in hCSF, we compared the growth of the wild type strain 106.66 with its capsule deletion mutant (106.66 Janus) as well as the wild type strain 51,114 L with its nonencapsulated spontaneous mutant, 51,114 S. Both the encapsulated strains 106.66 (serotype 6B) and 51,114 (serotype 19F) grew in hCSF to a higher OD than their nonencapsulated mutants (Fig. 2). This pattern was observed in hCSF from both patients tested. To check that this difference was not due to the bacteria with capsules being larger and therefore having a higher OD for the same number of bacteria than the nonencapsulated bacteria, the two phenotypes of strain 51,114 were grown again, in hCSF from a third patient, and plated out at 6 h. The CFU count was higher for the encapsulated strain (51,114 L) than its nonencapsulated mutant (51,114 S) confirming more bacterial growth when the bacteria possessed a capsule (Additional file 1: Figure S4).

### High carriage serotypes have a growth advantage in hCSF

**Fig. 3:**
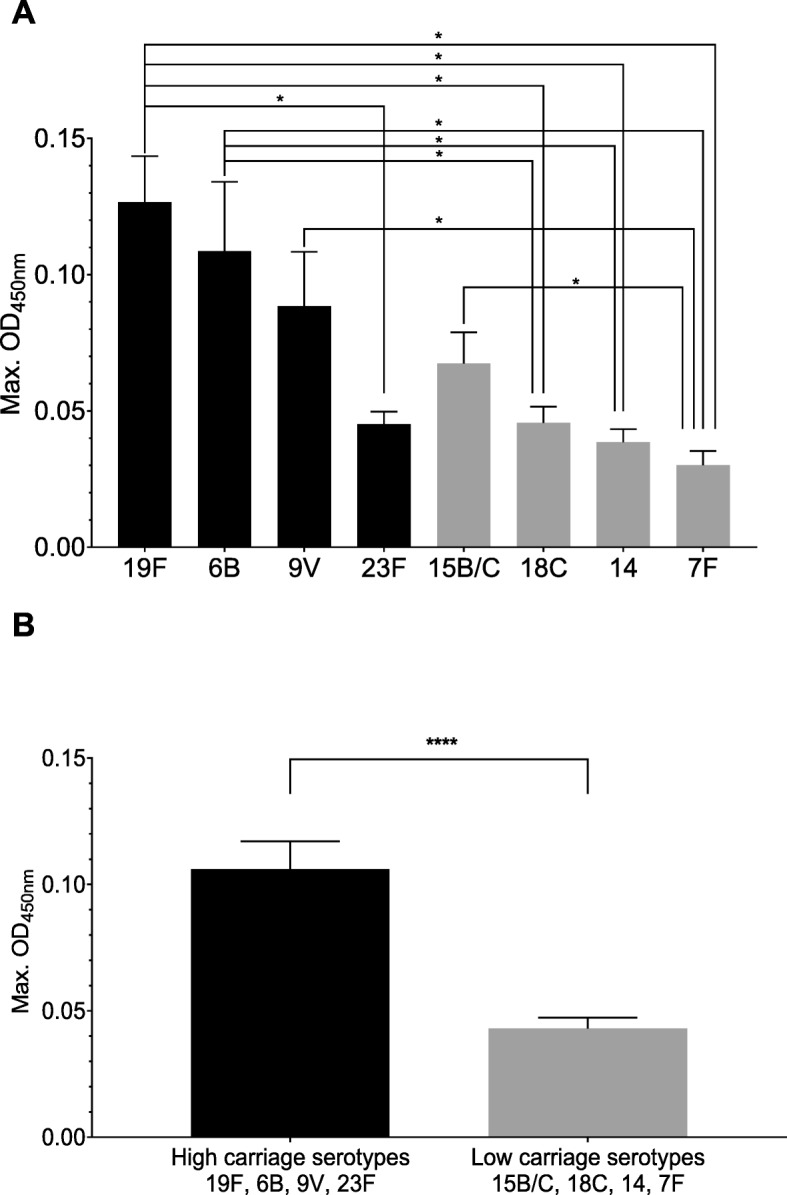
Growth maximum OD values of wild type strain 106.66 and capsule switch mutants in hCSF. **a** Maximum OD values in hCSF of wild type strain 106.66 and its capsule switch mutants. Black indicates high carriage prevalence, grey indicates low carriage prevalence according to [30], *p*-value < 0.04 (**b**) The mean of the maximum OD for all strains of high carriage serotypes (19F, 6B, 9 V and 23F) was greater than the mean of the maximum OD for all strains of the low carriage serotypes (15B/C, 18C, 14 and 7F), p-value < 0.0001. Error bar represent the standard error of the mean of three independent experiments

A previous publication showed that a 7F capsule switch mutant of strain 106.66 caused less severe disease in an animal model than its wild type serotype 6B parent [28]. In our study, switching the capsule to 7F significantly reduced OD_max_ in hCSF (Fig. 3a). When comparing the 6B to additional serotypes we found significant differences between serotypes 6B, 18C and 14; 19F and 18C; 23F, 14, and 7F; 9 V and 7F; 15B/C and 7F (*p*-value < 0.04). When pooling data for the four high carriage and four low carriage serotypes (defined according to our previous publication [29]), the low carriage serotypes reached significantly lower OD_max_ values in hCSF than the high carriage serotypes (Fig. 3b). No significant differences were observed when comparing the serotypes in BHI + FCS (Additional file 1: Figure S5). To confirm that differences in OD between serotypes in hCSF were really due to growth not bacterial size, CFU count was determined at 6 h and was higher for 6B and 19F than 14 and 7F (Additional file 1: Figure S6).

### Growth in hCSF correlates with case-fatality rate

**Fig. 4:**
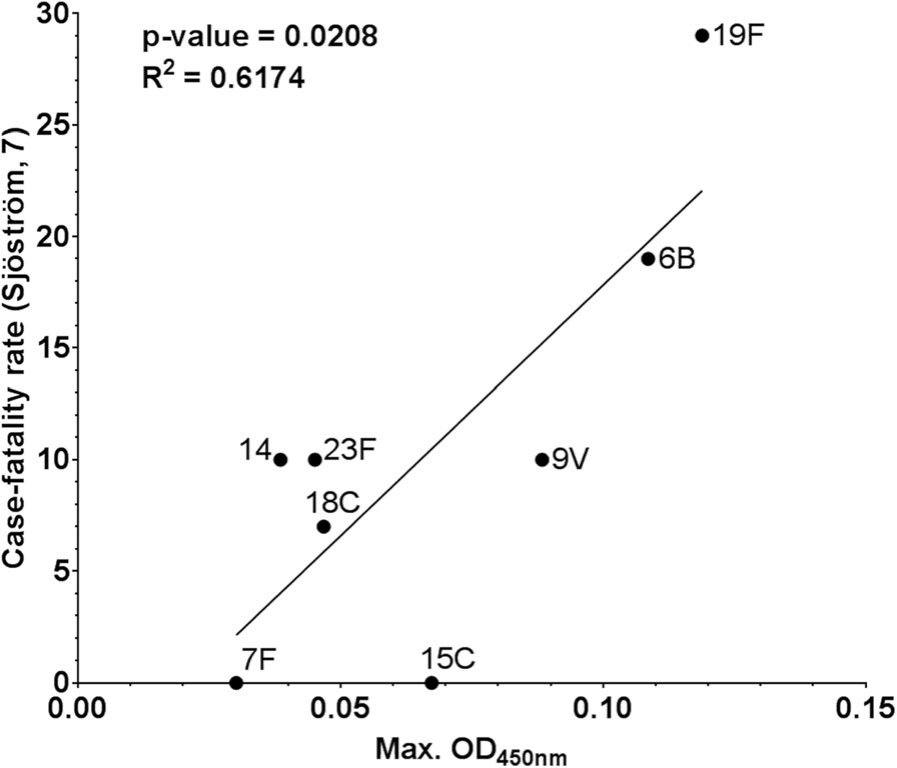
Correlation between case-fatality rate and maximum OD_450nm_ of different serotypes. For each serotype (6B *n* = 2, 7F n = 2, 14 *n* = 1; 9 V n = 2, 15B/C n = 1; 18C n = 2; 23F n = 1; 19F *n* = 3), the average OD_max_ reached in hCSF was plotted against the case-fatality rate obtained from a previous publication [7]. A positive correlation was found (R^2^ = 0.7645, p-value = 0.0100)

Since CFR is associated with serotype [8, 9] and we show that growth in hCSF is affected by capsule type; we looked whether growth in hCSF correlated with CFR by serotype. We observed a positive correlation between CFR of serotypes and the OD_max_ in hCSF (R^2^ = 0.6174, *p*-value = 0.0208) (Fig. 4).

### Capsule size is serotype-specific in hCSF

**Fig. 5:**
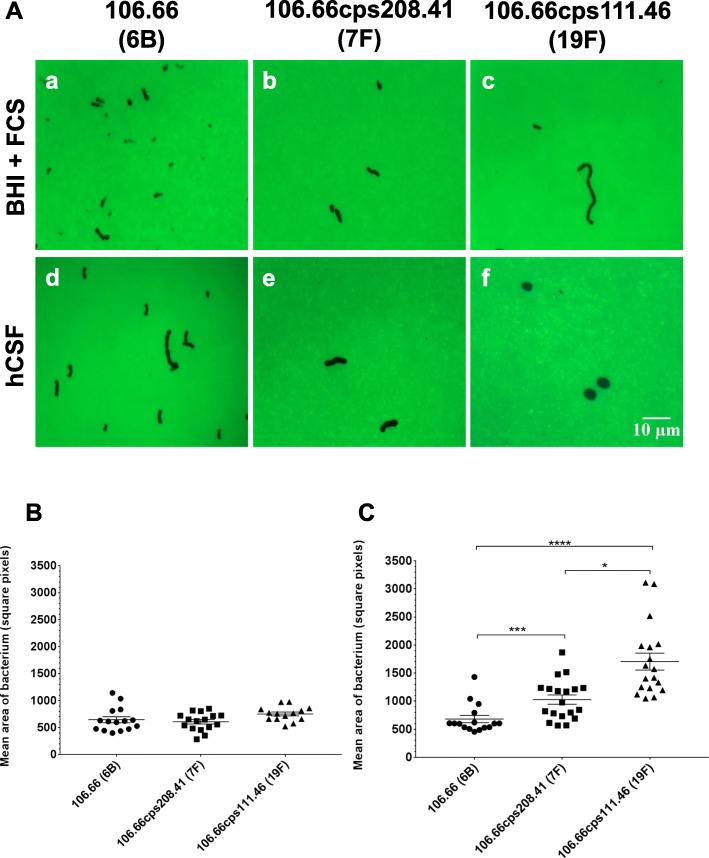
Capsule sizes of strains after 9 h of incubation in BHI + FCS or hCSF. **a** FITC-dextran exclusion images of wild-type strain 106.66 serotype 6B (a,d) and of capsule switch mutant 106.66cps208.41 (serotype 7F) (b,e) and 106.66cps111.46 (serotype 19F) (c,f) and after 9 h of incubation in BHI + FCS (a,b,c) and hCSF (d,e,f). All images are to the same scale, taken using a 100X objective, and the scale bar indicates 10 μm. **b** and **c** show the mean area of bacterium (square pixels) after 9 h of incubation of wild-type strain 106.66 serotype 6B compared to its capsule switch mutants 106.66cps208.41 serotype 7F and 106.66cps111.46 serotype 19F in **b** BHI + FCS or **c** hCSF. No significant difference was observed between any of the strains in BHI + FCS. Significant differences were found when comparing 106.66 with 106.66cps208.41 (*p*-value = 0.0008) and 106.66cps111.46 (*p*-value < 0.0001) and when comparing 106.66cps208.41 and 106.66cps111.46 (0.026). Each symbol represents one image containing at least 2 bacteria. Error bars represent the standard error of the mean of three independent experiments

We observed no difference in capsule size between any of the strains in BHI + FCS but in hCSF we observed a significant difference of capsule size between the serotype 6B wild type and the 7F and 19F capsule switch mutants (Fig. 5). The capsule size of both the 7F and the 19F capsule switch mutants were significantly greater in hCSF than the 6B serotype (Fig. 5c) and that of the 19F capsule switch mutant was significantly greater than that of the 7F (Fig. 5c).

## Discussion

We demonstrated that *S. pneumoniae* was able to grow in hCSF in vitro and that, contrary to the typical exponential growth pattern seen in BHI + FCS, the growth pattern in hCSF appeared as an extended straight line and lacked the autolysis phase. We speculated that the pattern may be due to the low nutritional composition of hCSF and because of the static in vitro environment where the hCSF is not replaced regularly as it would be under physiological conditions. However, during growth in low nutrient CDM with similar glucose level to hCSF, exponential growth and autolysis phase were still present. A previous study which analyzed the growth of *E. coli, S. aureus, L. monocytogenes, K. pneumoniae, S. epidermidis* as well as group B β-hemolytic streptococcus in hCSF hypothesized that hCSF inhibits the growth of these bacteria species [30]. We did not observe a reduction of growth of *S. pneumoniae* in a 1:1 mixture of hCSF and BHI + FCS compared with BHI + FCS alone. We therefore did not find evidence for pneumococcal inhibiting factors in hCSF, although we cannot rule out that any inhibitor diluted by half is below the concentration required to have a detectable effect. We speculate that the lack of autolysis may be due to low expression of either the *spxB* gene encoding pyruvate oxidase which produces hydrogen peroxide that may be a trigger for pneumococcal apoptosis [31] and/or the *lytA* gene which encodes the main autolysin degrading the cell wall and leading to pneumococcal lysis [32] but this has yet to be determined.

Presence of capsule enhanced growth in hCSF as following loss of capsule, growth was greatly reduced. This is in line with the literature which states that although nonencapsulated strains have been noted in IPD cases [33–35], these findings are very rare [36] and suggests that the capsule plays a fundamental role in survival and ability to grow in hCSF thereby contributing to meningitis.

Our results also showed that serotype influences the growth of *S. pneumoniae* in hCSF in vitro*.* Using one *S. pneumoniae* strain and comparing it to its mutants with insertion of different capsule genes to express different serotypes, we were able to study the effect of serotype in the absence of confounding effects of different genetic backgrounds. As we found previously in culture medium [30], we found that capsule type determines the growth phenotype in hCSF. The results also support another observation that capsule type rather than the genetic background is the main determinant of survival [37]. Furthermore, we noticed a pattern that serotypes categorized as invasive serotypes tended to have very poor growth in hCSF whereas serotypes categorized as high carriage serotypes tended to grow better in hCSF and that this correlates with serotype CFR. These results add to the evidence that the capsule plays an important role in severity of disease as high carriage serotypes are also often associated with more severe disease [8, 9]. The 19F serotype, which has repeatedly been associated with meningitis, reduced quality-adjusted life years, and increased CFR (reviewed in [8]), particularly showed a better ability to grow in hCSF than any of the other serotypes tested.

We also found significant differences in capsule size between 19F, 7F and 6B serotypes in hCSF. Of note, the 19F serotype, which is associated with severe disease [8, 9], and which grew best in hCSF, had the thickest capsule. Contrary to our expectations, however, serotype 7F also had a thicker capsule than 6B in hCSF even though 6B grew better than 7F as measured by both maximum optical density and number of colonies. In our previous publication, with these serotypes in an animal model, 6B appeared to have a thicker capsule than the 7F serotype. However, these differences may be explained due to physiological differences between rats and humans or because of the time point of sampling. A simpler capsule may be less metabolically demanding to synthesize allowing rapid growth and thick capsule in hCSF [30].

A limitation of our study is that, although growth was monitored in human CSF, all analyses were performed in vitro. We collected and froze the hCSF samples as soon as possible following lumbar puncture and did not refreeze samples but we cannot exclude the possibility that the composition of the hCSF changed during the time after removal from the patients. In vivo there would be a turnover of hCSF which would likely provide more nutrients to the bacteria. Therefore, with our in vitro experiments we may be underestimating the amount of pneumococcal growth in hCSF. This could be overcome by development of an in vitro system where the hCSF is replenished, if sufficient hCSF was available. Another major difference between our experiments and the situation in vivo is the absence of immune cells in our system. As meningitis progresses the number of immune cells in the hCSF might be expected to increase and any effect of this on bacterial growth and capsule thickness is not accounted for in our experiments. We deliberately chose to use hCSF from patients without inflammation to model early disease when pneumococci first reach the hCSF. However, it would also be interesting to study pneumococcal growth and capsule thickness in hCSF from patients with meningitis as we speculate that these may be affected by a nutritionally richer environment. We have made the assumption that the hCSF we have used is a model of healthy hCSF but the samples were taken from patients rather than healthy volunteers, although routine parameters were within normal ranges. Due to availability of samples, our experiments were limited to hCSF from 3 patients. Further testing in hCSF from more patients would be beneficial, although we note that the results we found were consistent between the hCSF of the three different patients.

A further limitation is the number of pneumococcal strains tested. It would be interesting to expand the study to further high and low carriage serotypes including those with large mucoid capsules such as serotypes 3 and 8 if the challenges of making capsule switch mutants of such serotypes could be overcome. However, we note that the pattern of growth, particularly the lack of autolysis was consistence for all strains tested.

## Conclusions

We found that in hCSF there was no autolysis and that serotype influenced growth and capsule size. Growth correlated positively with previously published serotype CFR ranges. Our findings contribute to the understanding of the role of serotype in pneumococcal disease, particularly meningitis. We further suggest that growth and capsule size in hCSF in vitro may be useful attributes to predict severity of disease caused by different serotypes to guide future vaccine design.

## Methods

### Bacterial strains

Swiss pneumococcal strain 106.66 (serotype 6B) is a clinical isolate from the nasopharynx of a child from a nationwide surveillance program collecting nasopharyngeal and invasive isolates [5, 38]. 106.66, its capsule deletion mutant (106.66 Janus) and several capsule switch mutants were used. 106.66 Janus and all capsule switch mutants are listed in Additional file 1: Table S1 (serotype 6B (*n* = 1), 7F (*n* = 2), 14 (n = 1), 9 V (n = 2), 15B/C (n = 1), 18C (n = 2), 23F (n = 1) and 19F (*n* = 3)). Production of the 106.66 capsule switch mutants and the 106.66 capsule deletion mutant (106.66 Janus) are described in a previous publication [30].

Additionally, a clinical isolate of serotype 19F (strain 51,114) recovered from the hCSF of an adult meningitis patient in 2017 from the South African GERMS national laboratory based surveillance program was provided by the National Institute for Communicable Diseases (NICD) in Johannesburg. This serotype was chosen due to its repeated association with meningitis and high CFR [8, 9]. Plating out of the frozen stock of South African serotype 19F strain 51,114 on Columbia sheep blood agar (CSBA) plates overnight (37 °C, 5% CO_2_) showed two colony phenotypes: large (51,114 L) and small colonies (51,114 S) with appearance of encapsulated and nonencapsulated pneumococci, respectively. The two phenotypes were separated and purified by three consecutive passaging steps where each time one single colony was picked and streaked on a CSBA plate. Separation as well as presence or absence of capsule was confirmed by serotyping and FITC-dextran exclusion assay (Additional file 1: Figure S1). Both phenotypes were confirmed to have the same genetic background (MLST 347) by multilocus sequence typing. This publication made use of the *Streptococcus pneumoniae* MLST website (https://pubmlst.org/ spneumoniae/).

For all strains, Quellung reaction was used to confirm serotype. The serotypes were categorized as low carriage / high invasive potential (7F, 14, 15B/C and 18C) or high carriage / low invasive potential (6B, 19F, 9 V and 23F) according to a previous paper [30].

### Human cerebrospinal fluid

Residual hCSF from three adult patients undergoing routine lumbar puncture in 2018 and 2019 due to idiopathic intracranial hypertension (non-inflammatory) was used for growth and capsule analysis. Lumbar puncture was performed by trained personnel. hCSF samples and related patient data were anonymized by the treating physician prior to any research use in accordance with the Swiss Human Research Law (Humanforschungsgesetz, HFG). Routine hCSF parameters were within normal ranges for all samples (glucose = 4.9 ± 1.6 mM; protein = 0.25 ± 0.07 g/l; cell count = 2 /ml). Samples were stored initially at 4 °C until collection and were aliquoted and frozen at − 80 °C within 48 h after lumbar puncture. All samples were used for analysis within 3 months of freezing. They were thawed 15 min before use. There was no pooling of CSF samples from different patients.

### Bacterial culture

Bacteria were stored at − 80 °C in Protect bacterial preservers (Technical Service Consultants, Heywood, U.K.). Bacteria were plated on CSBA and grown overnight in a 37 °C, 5% CO_2_ atmosphere. Three to 10 colonies were picked and used to inoculate tubes containing 5 ml brain heart infusion (BHI; Becton Dickinson and Company, le Pont de Claix, France) supplemented with 5% fetal calf serum (FCS; Biochrom KG, Berlin, Germany) (BHI + FCS). The culture was then incubated until OD_600nm_ reached 0.8–0.9. From this culture, a 250 μl sample was added to 750 μl fresh BHI + FCS. The culture was grown until OD_600nm_ reached 0.4 to ensure bacteria were in the exponential growth phase.

### Bacterial growth

When bacterial culture reached OD_600nm_ of 0.4, the culture was centrifuged for 5 min at 3000 g and the supernatant discarded. The culture was then re-suspended in 5 ml phosphate buffered saline pH 7.4 (PBS). Growth was assessed as described previously [30] with minimal changes described as follows. Sterile flat-bottomed 96-well microtitre plates (Nunclon Surface, Nunc, Denmark) were used based on the method of Brewster [39]. 8 μl of the PBS bacteria suspension was transferred into 200 μl of media (BHI + FCS, hCSF or CDM). CDM is a chemically defined medium with low nutritional value as defined in a previous publication [40] (with 4 mM glucose instead of 5.5 mM to mimic the hCSF glucose concentration). For growth in BHI + FCS and CDM, each strain was used to inoculate three wells per experiment. For growth in hCSF, due to limited amount of available hCSF, one well per bacterial strain was inoculated per experiment. The plate was then incubated at 37 °C and OD_450nm_ measured using a VERSAmax microplate reader (Molecular Devices). SoftMax Pro Software, version 5.3 was used to record measurements. OD was measured at 30 min intervals over 40 h with 5 s of automatic shaking preceding each reading. Condensation was prevented by pre-treating the plate lid with 3 ml 0.05% Triton X-100 in 20% ethanol [30]. All experiments were performed three times on three separate days. Differences in growth between serotypes were assessed by comparing maximum OD_450_ (OD_max_) over 40 h.

To determine colony forming units (CFU), dilutions of bacterial cultures were plated onto CSBA plates and cultured overnight at 37 °C, 5% CO_2_ atmosphere and colonies counted the following day.

### Correlation of serotype specific growth in hCSF with CFR

To analyze whether the ability to grow in hCSF in vitro correlated with disease severity we plotted the OD_max_ that each serotype reached in hCSF or BHI + FCS over 40 h against the corresponding serotype case-fatality rate (CFR). The CFR was taken from a previous publication by Sjöstrom K, et al. (2006) [7] in which CFR was calculated based on data collected from 494 adult patients with invasive pneumococcal disease.

### Capsule size

The FITC-dextran method of Gates et al. [41], in which the zone of exclusion is measured, was used for capsule size analysis. Bacteria were cultured as described above. After reaching OD_600nm_ of 0.4 the culture was centrifuged at 3000 g for 5 min. The supernatant was discarded and the pellet re-suspended in 5 ml PBS. A sample of 50 μl bacterial suspension was used to inoculate 600 μl of BHI + FCS or hCSF. The tubes were incubated in a water-bath at 37 °C. FITC-dextran exclusion assay was performed at time 0 and 9 h after incubation. To perform the FITC-dextran assay, 10 μl of the bacteria culture was removed and mixed with 2 μl FITC-dextran (2000 kDa, Sigma; 10 mg/ml in water). The mixture was pipetted onto a microscope slide and a coverslip was applied. A Zeiss Axio Imager M1 fluorescence microscope with a 100 x objective was used to view the slides. The Zeiss AxioCam HRc camera was used for photographing the slides. Capsule size was measured from at least 5 randomly selected bacterial cell bodies over five images per group of each of the three independent images, adding up to a total of 15 images per group.

We performed FITC-dextran exclusion assays of wild type strain 106.66 serotype 6B, its 7F capsule switch mutant (106.66cps208.41) and a capsule switch mutant of 19F serotype (106.66cps111.46) due to the association of serotype 19F with meningitis and a high CFR [8] and because it had the highest average OD_max_ value in hCSF.

### Statistics

Statistical analysis and graphs were performed using the GraphPad Prism software (version 7.04, GraphPad Software). The same software was used for linear regression analysis for which a *p*-value, two-tailed, of ≤0.05 was considered significant. The comparisons of OD_max_ were performed using a non-parametric Mann Whitney, two-tailed comparison test. A value of *p* ≤ 0.05 was considered significant.

## Supplementary information

**Additional file 1: Table S1.** List of 106.66 capsule switch mutants used in experiments [1]. **Figure S1.** Capsule thicknesses of strains 106.66 and 51,114 with and without capsule in BHI + FCS. **Figure S2.** Growth pattern of *S. pneumoniae* wild type strain 106.66 and 106.66 capsule switch mutants representing high carriage serotypes (A, C) and low carriage serotypes (D, B) in BHI + FCS^1^ (A, B) and hCSF^2^ (C, D) over 40 h. **Figure S3.** Growth of *S. pneumoniae* wild type strain 106.66 in BHI + FCS, CDM^3^ [2], hCSF and a 1:1 mix of hCSF and BHI + FCS over 40 h. **Figure S4.** Colony forming units (CFU) after 6 h of growth in human CSF (hCSF) for South African strain 51,114 L (serotype 19F) and its spontaneous capsule loss mutant 51,114 S. **Figure S5.** Maximum OD values of wild type 106.66 and capsule switch mutants in BHI + FCS. **Figure S6.** Colony forming units (CFU) after 6 h of growth in human CSF (hCSF).

## Data Availability

All data generated or analysed during this study are included in this published article and its supplementary information files.

## Abbreviations

BHI: Brain heart infusion
CDM: Chemically-defined medium
CFR: Case-fatality rate
CFU: Colony forming units
CSBA: Columbia sheep blood agar
FCS: Fetal calf serum
hCSF: Human cerebrospinal fluid
IPD: Invasive pneumococcal disease
MLST: Multi-locus sequence type
NVT: Non-vaccine type
OD: Optical density
PBS: Phosphate-buffered saline
PCV13: Pneumococcal conjugate vaccine 13-valent
PPSV: Pneumococcal polysaccharide vaccine 23-valent
VT: Vaccine type
