# Supplementary material for: Pneumococcal serotype determines growth and capsule size in human cerebrospinal fluid
Source: BMC Microbiol. 2020 Jan 20;20:16. doi: 10.1186/s12866-020-1700-7 (PMC6971925; doi:10.1186/s12866-020-1700-7)
Supplement: Supplementary file 1 — Additional file 1: Table S1. List of 106.66 capsule switch mutants used in experiments [1]. Figure S1. Capsule thicknesses of strains 106.66 and 51,114 with and without capsule in BHI + FCS. Figure S2. Growth pattern of S. pneumoniae wild type strain 106.66 and 106.66 capsule switch mutants representing high carriage serotypes (A, C) and low carriage serotypes (D, B) in BHI + FCS1 (A, B) and hCSF2 (C, D) over 40 h. Figure S3. Growth of S. pneumoniae wild type strain 106.66 in BHI + FCS, CDM3 [2], hCSF and a 1:1 mix of hCSF and BHI + FCS over 40 h. Figure S4. Colony forming units (CFU) after 6 h of growth in human CSF (hCSF) for South African strain 51,114 L (serotype 19F) and its spontaneous capsule loss mutant 51,114 S. Figure S5. Maximum OD values of wild type 106.66 and capsule switch mutants in BHI + FCS. Figure S6. Colony forming units (CFU) after 6 h of growth in human CSF (hCSF). [file 12866_2020_1700_MOESM1_ESM.docx]

**Supplementary Material**

**Table S1:** List of 106.66 capsule switch mutants used in experiments ([1](#_ENREF_1))

| **106.66 Capsule Switch Mutants** | |
| --- | --- |
| **Mutant** | **Serotype** |
| 106.66cps106.66 | 6B |
| 106.66cps208.41 | 7F |
| 106.66cpsB109.15 | 7F |
| 106.66cpsB101.77 | 14 |
| 106.66cps201.38 | 9V |
| 106.66cps109.74 | 9V |
| 106.66cps207.31 | 15B/C |
| 106.66cps307.14 | 18C |
| 106.66cpsB112.27 | 18C |
| 106.66cps103.57 | 23F |
| 106.66cps108.34 | 19F |
| 106.66cps111.46 | 19F |
| 106.66cps201.47 | 19F |
| 106.66 Janus | non-encapsulated |

**Figure S1: Capsule thicknesses of strains 106.66 and 51114 with and without capsule in BHI+FCS** (A) FITC-dextran exclusion images of wild-type strain 106.66 serotype 6B (a), non-encapsulated strain 106.66 (106.66 Janus) (c), clinical isolate 51114 serotype 19F (b) and clinical isolate 51114 after spontaneous capsule loss (spon. capsule loss) (d). All images are to the same scale, original magnification is 100X, and the scale bar indicates 10 µm. (B) Mean area of bacterium (square pixels) in 5 ml BHI+FCS of wild-type strain 106.66 serotype 6B, non-encapsulated strain 106.66 (106.66 Janus), clinical isolate 51114 serotype 19F and clinical isolate 51114 after natural capsule loss. Error bars represent the standard error mean of three independent experiments


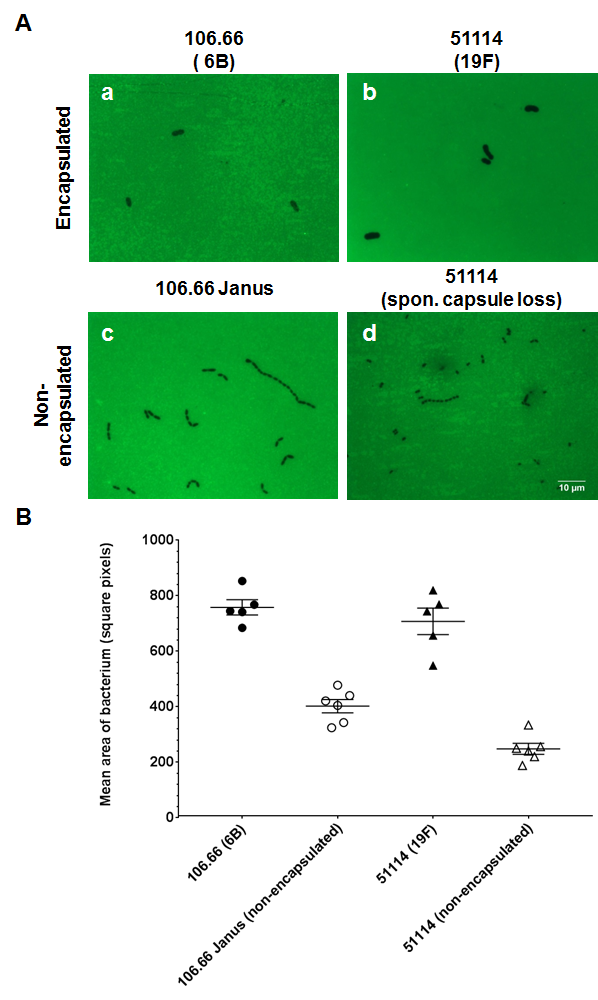


**Figure S2: Growth pattern of S. pneumoniae wild type strain 106.66 and 106.66 capsule switch mutants representing high carriage serotypes (A, C) and low carriage serotypes (D, B) in BHI+FCS^[[1]](#footnote-1)^ (A, B) and hCSF^[[2]](#footnote-2)^ (C, D) over 40 hours.** Each data point represents an average of minimum 3 independent experiments.

**(A) (B)**

**(C) (D)**

**Figure S3: Growth of** ***S. pneumoniae* wild type strain 106.66 in BHI+FCS, CDM^[[3]](#footnote-3)^ (**[**2**](#_ENREF_2)**), hCSF and a 1:1 mix of hCSF and BHI+FCS over 40 hours**. Each data point represents an average of minimum 3 independent experiments for all growth curves except the 1:1 hCSF and BHI+FCS mix due to limited amount of available hCSF.

**Figure S4: Colony forming units (CFU) after 6 hours of growth in human CSF (hCSF) for South African strain 51114 L (serotype 19F) and its spontaneous capsule loss mutant 51114 S.** More colonies were obtained for the encapsulated than the nonencapsulated pneumococci following growth in hCSF. Results show the mean of three independent experiments performed on three different days, each in triplicate. Error bars indicate the SEM.

**Figure S5: Maximum OD values of wild type 106.66 and capsule switch mutants in BHI+FCS** (A) individual maximum OD values of capsule switch mutants with black bars representing high carriage serotypes (6B, 9V, 19F, 23F) and gray bars representing low carriage serotypes (14, 15B/C, 18C, 7F). (B) Maximum OD values in BHI+FCS of serotypes pooled according to a previous publication ([1](#_ENREF_1)) into serotypes representing high carriage serotypes and low carriage serotypes. Error bars represent the standard error of the mean of three independent experiments.

**(A)**

**B/C**

**(B)**

**Figure S6: Colony forming units (CFU) after 6 hours of growth in human CSF (hCSF)** (A) *S. pneumoniae* wild type strain 106.66 (serotype 6B) and its capsule switch mutants 106.66cps111.46 (19F), 106.66cpsB101.77 (14) and 106.66cps208.41 (7F). More colonies were obtained for the high carriage serotypes (represented in black) than the low carriage serotypes (represented in grey). Results show the mean of three independent experiments performed on three different days, each in triplicate. Error bars indicate the SEM. (B) When data from (A) is pooled the difference between the high (6B and 19F) and low (14 and 7F) carriage serotypes is significant (*p=0.0411).

(A)

(B)

**REFERENCES**

1. Hathaway LJ, Brugger SD, Morand B, Bangert M, Rotzetter JU, Hauser C, et al. Capsule type of Streptococcus pneumoniae determines growth phenotype. PLoS pathogens. 2012;8(3):e1002574. PubMed PMID: 22412375. Pubmed Central PMCID: 3297593.

2. Schaffner T, Hinds J, Gould K, Wüthrich D, Bruggmann R, Küffer M, et al. A point mutation in cpsE renders Streptococcus pneumoniae nonencapsulated and enhances its growth, adherence and competence. BMC Microbiology. 2014;14:210-22.

1. Brain heart infusion broth (BHI) supplemented with 5 % fetal calf serum (FCS) (BHI+FCS) [↑](#footnote-ref-1)
2. Human cerebrospinal fluid (hCSF) [↑](#footnote-ref-2)
3. Chemically defined media with low nutritional value as defined in a previous publication (2) (with 4 mM glucose instead of 5.5 mM to mimic the hCSF glucose concentration). [↑](#footnote-ref-3)
